# The Promise and Perils of Pre-Publication Review: A Multi-Agent Simulation of Biomedical Discovery Under Varying Levels of Review Stringency

**DOI:** 10.1371/journal.pone.0010782

**Published:** 2010-05-26

**Authors:** Jeff Shrager

**Affiliations:** 1 Symbolic Systems Program, Stanford University, Stanford, California, United States of America; 2 CollabRx Inc., Palo Alto, California, United States of America; Aarhus University, Denmark

## Abstract

The Internet has enabled profound changes in the way science is performed, especially in scientific communications. Among the most important of these changes is the possibility of new models for pre-publication review, ranging from the current, relatively strict peer-review model, to entirely unreviewed, instant self-publication. Different models may affect scientific progress by altering both the quality and quantity of papers available to the research community. To test how models affect the community, I used a multi-agent simulation of treatment selection and outcome in a patient population to examine how various levels of pre-publication review might affect the rate of scientific progress. I identified a “sweet spot” between the points of very limited and very strict requirements for pre-publication review. The model also produced a u-shaped curve where very limited review requirement was slightly superior to a moderate level of requirement, but not as large as the aforementioned sweet spot. This unexpected phenomenon appears to result from the community taking longer to discover the correct treatment with more strict pre-publication review. In the parameter regimens I explored, both completely unreviewed and very strictly reviewed scientific communication seems likely to hinder scientific progress. Much more investigation is warranted. Multi-agent simulations can help to shed light on complex questions of scientific communication and exhibit interesting, unexpected behaviors.

## Introduction

Modern science is a highly collaborative, community practice. Scientists often collaborate directly, and the scientific literature supports building upon previous work over time. New electronic publishing technologies, such as e-journals, forums, and blogs, offer the enticing possibility of highly efficient instant publication, rather than authors having to go through the cumbersome processes of classic peer review and long publication cycles. In the extreme, scientists may put their lab notebooks or straight-off-the-instrument data online. Of course, unreviewed publication is a venerable tradition both in classic and modern science; scientists often publish in books, editorials, and other venues that are not peer reviewed; some have even run their own presses, like Benjamin Franklin did. But the ease and speed with which one can publish on the Web, combined with the critical role that Web search has taken as the first resource for nearly all scientific scholarship, raises new possibilities and the potential for new problems. Nielsen [1] eloquently describes the possibilities: “The Internet offers us the first major opportunity [to create] a conversational commons for the rapid collaborative development of ideas.” On the other hand, the Internet poses problems with information quality control. Shrager et al. [2] suggest that reducing pre-publication review requirements may lead to a decrease in the average quality of the information stream; high-quality information will be less clearly marked, and information may be quickly posted and therefore less stable due to revisions. With the vast amount of information available, scientists could act based on unreliable information. Although some rapidly-published results are available for post-publication review, readers often miss these types of reviews. Moreover, as the pace of information distribution in the community speeds up, a vicious cycle may result that pushes researchers to act faster to ensure they publish first, potentially reducing their capacity to check their own results. A Web-based review process must be carefully designed to allow for easy filtering of publications based upon their review type and quality.

Ominous as this scenario may sound, it is not entirely clear that these changes are for the worse. The rapidity with which results are posted and shot down may balance out a decrease in the quality of published results. Thus even if the signal-to-noise ratio of published results is reduced, the search among alternative hypotheses could take place so much faster that this balances out the time wasted by the community in following up published results that turn out to be invalid.

Whether, and under what model, pre-publication review should take place is no mere academic question; scientists encounter this struggle daily. Pöschl [3] recently observed that “shorter articles and an increasing number of publications have resulted in the scientific information market being flooded by journal articles, preprints and proceedings with little or no quality control.” Moreover, unreviewed (or lightly reviewed) material is rapidly leaking into the supposedly validated scientific literature without any indication of the level of review it has received. For example, the online journal *PLoS Currents: Influenza* [4] is “moderated by an expert group of influenza researchers, but in the interest of timeliness, does not undergo in-depth peer review.” Yet the abstracts from this journal appear in PubMed–a widely accepted source for validated biomedical publication–with no indication they have not undergone in-depth peer review. By contrast, arXiv [5] provides quality control through two levels of review, endorsement and moderation, and will not certify a paper until it has appeared in a peer-reviewed journal.

With the current state of the review process, and new publication paradigms rapidly upon us, now is the time to question the impact that a flood of lightly reviewed scientific publications could have on scientific progress. For example, the first volume of the newly minted *Journal of Participatory Medicine* (JPM) [6] launched online in October 2009, featured an essay wherein Richard W. Smith [7], former editor of the *British Medical Journal*, encouraged the readership of JPM to help design a new alternative to traditional peer review: “[We] don't yet have a clearly articulated alternative to peer review, but this is your chance to ‘join the revolution’ and together with the editors devise a better system for this journal.” Smith asks his readers to consider a model under which new data is put online immediately, with reviewer and editor comments available after online publication. But again, our modern Web experience suggests that post-publication commentary of this sort often goes largely unnoticed. Indeed, Google's Page Rank algorithm ranks documents primarily based upon the number of citations, pushing papers, with more citations higher in rank even if the citations are not complementary.

Although Web-based publication has not yet resulted in the chaos envisioned by Shrager, et al., we are rapidly headed for a world of increasingly lightly reviewed, post-reviewed, or unreviewed scientific publications. In his exhortation, Smith asks the readers to contribute their thoughts based on evidence. There are various sorts of evidence that might bear on this problem. In the present paper, I employ a multi-agent, computational simulation to explore what impact self-publication and lightly reviewed, post-reviewed, or unreviewed publication might have on the progress of science. Multi-agent simulations have been used to good effect in a wide variety of domains [8], but have rarely been used to model the scientific community. Payette [9] developed a multi-agent simulation based upon the hypothesis that scientists earn the respect of their peers through sharing ideas with their collaborators and competitors. In this model, scientists “propagate their ideas [and] acquire credit, i.e., the consideration of their peers, with whom they are both competing and collaborating.” Payette's simulated scientists operate in a social network with their students and collaborators while writing peer-reviewed articles that allow them to share their ideas with the whole community. Each idea is assigned a real-value of ‘empirical adequacy’, although the scientists do not have direct access to this value and must approximate it using tests. When ideas are transmitted from one scientist to another, noise can be introduced into the transmission, and the efficiency of the system is measured by how close the subjective ratings assigned by the agents to ideas come to the real, objective correctness of the idea.

My model employs a similar multi-agent approach to the question of whether pre-publication review accelerates or hinders the progress of science. I simulate the health of a community with a population of 1000 persons and realistic probabilities of getting a progressive, fatal disease, such as cancer. In this model, patients with cancer are treated with the best available published treatment, and observe the population after 100 years. Like Payette's model, the present model contains ubiquitous and inherent measurement noise; it is impossible for any agent to directly observe the true state of health of themselves or of any other agent. This will come into play in trying to understand how the predictions and observed results arise. The main variable I explore here is the stringency of the requirements for publication, modeled here as the number of sequentially observed improvements in a particular patient's health status; that is, approximately: replications required to publish. I call this ‘HMSDCALE’ and abbreviate is as: ‘@N’, where N is the minimum number of sequentially observed improvements required to publish. The present experiments vary HMSDCALE in the range of @1 (very weak replication requirements) through @12 (very strict replication requirements). I predict that both extremes on the scale will be inferior to mid-range values, and the population size will decrease over the course of one-hundred years; however, the explanation for this downward trend is different at each end of the scale. At the weak end (near @1), nonsense observations will rapidly fill the literature, misleading the community to adopt improper treatments, whereas at the strict end (near @12) almost nothing will be published because measurement noise makes it almost impossible to achieve the required number of sequentially observed improvements in the patient's health. In this latter scenario, treatment selection has no basis in published evidence and is therefore effectively random. Both of these cases should have adverse consequences for the overall health of the population. More details of the model appear in the Materials and Methods section below.

## Results

**Figure 1:**
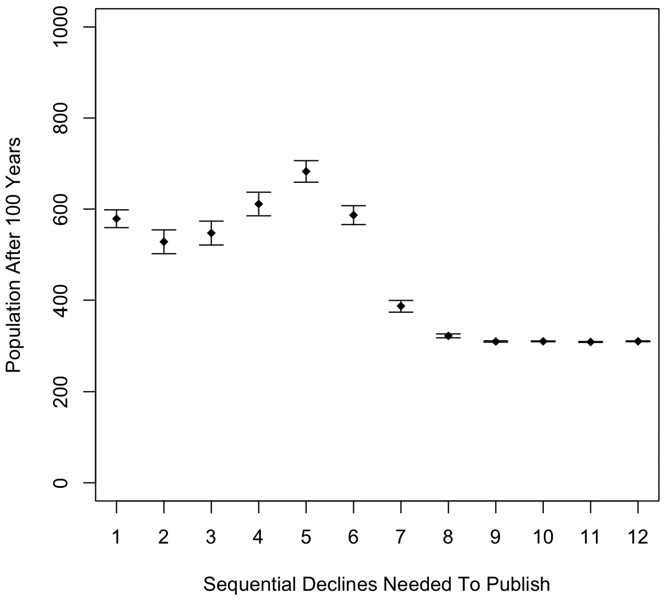
Populations after 100 years of simulation across increasing values of HMSDCALE (@1-12). Standard error (s.e.) bars are over 250 replications with different random seeds. (The s.e. bars are so small as to be nearly invisible at high values of HMSDCALE.)

Figure 1 depicts the number of patients that remain alive at the end of 100 years of simulation across a range of HMSDCALE values from @1 through @12. These data are accumulated over 250 replicated runs with different random seeds. The error bars represent standard errors (s.e.). Overall, this result is highly significant (F(11,2988) = 69.43, p∼ = 0.00), but further analysis is required in order to confirm or refute the theory put forward above.

First, note that the population falls off steeply at the high end of HMSDCALE values. This happens because measurement error makes it very unlikely to observe high numbers of sequential declines, even in a patient whose health is improving monotonically. As a result, very few effective treatments are published in this upper range of HMSDCALE. There is a fairly smooth decline from @5 through @8, reaching a minimum of about 300 people alive after 100 years. Not surprisingly, this is about the same result obtained if random treatments are given. In post-hoc ANOVA analysis (by Tukey HSD), there are no significant differences between @9-@12, but @7-@12 all show highly significant differences (p<.001) from the other values (@1-@6). The HMSDCALE at @7 is nearly significantly different from each value in @9 to @12 (9v7∼ = 0.08, 10v7∼ = 0.08, 11v7∼ = 0.07, 12v7∼ = 0.08). Of the remaining post-hoc comparisons, the only significantly different ones were as follows: 5v1∼ = 0.001, 4v2∼ = 0.03, 5v2∼ = 0.00, 5v3∼ = 0.00, and 6v5<0.01.

Notice that comparing @1 to @5, the final population increases significantly (p∼ = 0.001) followed by the decline described above. An increase from @1 through some medium point is roughly what was expected. This supports the hypothesis that lightly reviewed publication is inefficient, and some moderate level of publication stringency is superior to either very weak or very strong levels. Five simulated years of observed, monotonic improvement in disease state is approximately the “sweet spot” for pre-publication replication requirements in this particular simulation regimen.

Interestingly, the values between @1 and @5 decrease somewhat. Although @1 is not significantly different from @2–4, there is a difference between @2 and both @4 and @5, and a difference greater than the standard error between both @2 and @3 compared to @5, yielding post-hocs of 4v2∼ = 0.03, 5v2∼ = 0.00, and 5v3∼ = 0.00. This u-shaped result was not expected, so I explored it in more detail. A closer look at the standard errors suggests the data in this range is essentially bimodal; either the population discovers the right treatment, publishes the results fairly rapidly, and essentially everyone survives; or they never discover it, resulting in approximately the same lower population as in the higher values on the scale. What is changing in the @1–5 range is the number of the 250 runs that land in each of these regimens.

One can more clearly observe this bimodality by splitting the data shown in Figure 1 into the high-end and low-end of the scale. To show this more easily, I have split the data at a remaining population of 800. Table 1 gives the total number of runs of the 250 where the population after 100 years was greater than or equal to 800 as well as the associated means and standard errors (s.e). This division significantly reduces the s.e. for almost all of the data, from a s.e. around 25 in the @2–5 range down to s.e. in the low single digits. This supports the hypothesis of strong bimodality in the data, and can be seen by looking at any of the dynamics in the @1-@6 range. Notice that the number of people remaining after 100 years is around 170 for all of the <800 cases in @2–5, which is around the level of the null treatment; that is, all patients are always treated with a drug that has no effect. This suggests that once someone randomly discovers a good treatment, everyone still alive at that point is saved. If this happens early in the simulation, almost everyone is saved. But unless and until the correct treatment is discovered and published, everyone is being treated randomly, and with mostly poor treatments. The HMSDCALE parameter simply pushes the point in time when this discovery takes place to a later date, making it harder to discover a good treatment but also improving the performance of the treatment once discovered. At low HMSDCALE values, incorrect treatments are published and have little utility, whereas, as the HMSDCALE rises up to a point around @5 or @6, the quality of the published treatments is improved. However, these discoveries come later in the run. After about @6, publication starts to be so difficult that eventually nothing is published at all and treatments are essentially random. And indeed, the level reached, about 300, is about the same as random treatment. Note that random treatment is slightly superior to null treatment as the variation inherent in the random approach means the treatments may be either more or less effective than the null. The parameters of the present model lean slightly toward more good than poor treatments. The combination of these phenomena creates the observed complex dynamics.

**Table 1:** Statistics from 250 runs split between a final population of > = 800 v.<800 after 100 years.

@>	combined Mean	combined se	n> = 800	> = 800 mean	> = 800 se	n<800	<800 mean	<800 se
1	578.82	19.53	86	966.79	4.86	164	375.37	11.98
2	528.19	26.16	109	997.33	0.56	141	165.52	1.86
3	547.34	26.21	115	995.00	0.68	135	166.01	2.10
4	611.16	25.94	135	988.50	1.03	115	168.20	2.26
5	682.69	23.72	162	957.69	2.37	88	176.45	2.88
6	586.73	20.74	105	898.78	5.18	145	360.76	20.58
7	387.00	12.34	18	884.61	13.12	232	348.39	9.28
8	322.69	4.34	1	803.00	0.00	249	320.76	3.90
9			0			250	310.32	1.50
10			0			250	310.63	0.92
11			0			250	309.24	0.92
12			0			250	310.72	0.93

## Discussion

Although limited in scope and applicability, these results suggest that different levels of stringency in peer review may accelerate or hinder scientific progress. Too strict of a review requirement (represented here by the HMSDCALE parameter) can prevent sharing of valid treatments, but too weak of a requirement drowns good results in a sea of bad treatments. Moreover, unexpected, non-linear complexities appear between the point of too little review (@1-4) and the “sweet spot” of five observed sequential improvements (@5).

These results certainly have not answered the specific question of exactly how much pre-publication review is too much or too little. For example, it is unlikely that doctors should wait five years for the improvement of a cancer patient before publishing. (Coincidentally, cancer-free for five years is exactly what is considered a “cure” in oncology.) However, I have shown that a relatively simple model can suggest general principles and reveal interesting, complex phenomena that invite further analysis. In this way, multi-agent models offer a new instrument for investigation of the complex social structures that are modern science.

## Materials and Methods

Supporting file Text S1 contains the complete, self-contained Common Lisp code for the model, along with the parameter values that produced the results presented here.

Agents, that is, patients, doctors, or patient–doctor units, operate in an environment of a single disease (e.g., cancer) and numerous potential treatments. A population of patients (here 1000) stochastically becomes ill, for example, at a rate of 0.01 of the population per year. When the disease is initially diagnosed in a given person, it has a real-valued level (0.1), and progresses each year by a fixed factor (1.2×) until it reaches a death threshold (0.5). At this point the individual is removed from the population. Patients visit the doctor once each year and either continue with or change their current treatment. The model is usually run for 100 simulated years, and I conduct 250 replication runs with different random seeds. The available drugs used to treat the disease take on a range of multiplicative effectiveness, with an effectiveness of 1.0 having no effect (i.e., a null treatment). Values smaller than 1.0 denote beneficial effects (i.e., reducing the level of the disease), and those greater than 1.0 denote undesired side effects (i.e., accelerating towards the death threshold). For example, if a person is diagnosed at 0.1 and is treated for three years with a null drug (effectiveness = 1.0) and a disease progression factor of 1.2, he or she will have a disease level of ∼0.173 in the third year. If he or she then begins treatment with a drug that has a treatment effectiveness of 0.8, his or her next disease level will be 0.173×1.2×0.8 = 0.166, then 0.159, etc., and this patient will eventually be “cured” if he or she remains on this drug; that is, the disease level will approach, although never quite reach, 0.0.

In the present model, every person employs the same decision algorithm. When initially diagnosed, the patient gets the best drug reported in the literature at the time. This method is described below. If the disease has not apparently improved by the patient's annual visit to the doctor, that is, the measured disease level has not reduced, this patient is again treated with the current best drug in the literature, which will almost certainly have changed since the patient's last visit. Note that on each visit to the doctor the patient's actual state of health can only be measured with a standard deviation of 2.0, so that the actual state of the patient's health is not directly observable, similar to Payette's model.

Agents in the present model interact with one another via publication. At each annual treatment, either the patient or his or her doctor may publish the result in the collective literature, which is a simple, chronological stack. Results are only published after the patient has a number of sequential improvements while taking the same drug, as determined by the HMSDCALE parameter. Recall that patient improvement inherently contains error, so that an error on a single measurement could cause a misinterpretation of the patient's state. I use the notation “@N” to indicate an HMSDCALE value of N. The range between the @1 and higher values of HMSDCALE models the range between no pre-publication review, that is no requirement of replication where everything that shows any promise is published into the literature, and a very stringent requirement of observing monotonic improvement over many sequential observations. An HMSDCALE of @1 is close to, but not quite literally publishing everything because in a true publish-everything setting publication would take place regardless of whether the patient's health is improving. I do not currently model “negative results”–that is, a well-powered study that shows no effect–nor publication of poor outcomes–that is, replicable declining health status rather than improving health status.

Modeling statistics were carried out by one-way ANOVA and Tukey HSD post-hoc tests.

## Supporting Information

Text S1 Stand Alone Simulation Common Lisp Code.
(0.03 MB TXT)
